# Early diagnosis of neural impairment in seropositive leprosy household contacts: The experience of a reference center in Brazil

**DOI:** 10.3389/fmed.2023.1143402

**Published:** 2023-03-13

**Authors:** Diogo Fernandes dos Santos, Leonardo Peixoto Garcia, Isabella Sabião Borges, Thales Junqueira Oliveira, Douglas Eulálio Antunes, Andrea De Martino Luppi, Isabela Maria Bernardes Goulart

**Affiliations:** ^1^National Reference Center for Sanitary Dermatology and Leprosy, Clinics Hospital, School of Medicine, Federal University of Uberlândia (UFU), Uberlândia, Brazil; ^2^Postgraduate Program in Health Sciences, School of Medicine, Federal University of Uberlândia (UFU), Uberlândia, Brazil

**Keywords:** leprosy, Hansens’ disease, mycobacterium leprae, household contacts, neural impairment, peripheral neurophaty

## Abstract

**Introduction:**

Leprosy is an infectious disease that remains with a high number of new cases in developing countries. Household contacts have a higher risk for the development of the disease, but the neural impairment in this group is not well elucidated yet. Here, we measured the chance of occurrence of peripheral neural impairment in asymptomatic leprosy household.

**Methods:**

Contacts who present anti-PGL-I IgM seropositivity, through electroneuromyography (ENMG) evaluation. We recruited 361 seropositive contacts (SPC) from 2017 to 2021, who were subjected to an extensive protocol that included clinical, molecular, and electroneuromyographic evaluations.

**Results:**

Our data revealed a positivity of slit skin smear and skin biopsy qPCR of 35.5% (128/361) and 25.8% (93/361) respectively. The electroneuromyographic evaluation of the SPC showed neural impairment in 23.5% (85/361), with the predominance of a mononeuropathy pattern in 62.3% (53/85). Clinical neural thickening was observed in 17.5% (63/361) of seropositive contacts, but among the individuals with abnormal ENMG, only 25.9% (22/85) presented neural thickening in the clinical exam.

**Discussion:**

Ours results corroborates the need to make the approach to asymptomatic contacts in endemic countries more timely. Since leprosy in its early stages can present an indolent and subclinical evolution, serological, molecular, and neurophysiological tools are essential to break the disease transmission chain.

## 1. Introduction

Leprosy is a chronic disease due to infectious by *Mycobacterium leprae (M. leprae)* that remains an important health problem in developing countries, such as India and Brazil, because of the late diagnosis and a high number of new cases (1). This bacillus has a slow replication rate with a long incubation period and infects especially peripheral nerves and skin (2).

The World Health Organization classifieds leprosy into paucibacillary (PB) or multibacillary (MB) forms according to the number of skin lesions and the slit skin smear aiming treatment protocols (3). In clinical practice, the classification of Ridley and Jopling is also used (4), classifying patients into five clinical forms: tuberculoid, borderline-tuberculoid, borderline-borderline, borderline-lepromatous and lepromatous.

Besides these clinical forms and the operational classification of WHO, a major challenge in this chronic disease is the definition of subclinical infection or latent leprosy. Even in the absence of symptoms, *M. leprae* is replicating and invading the host tissues (5), and biomarkers for the infection as anti-phenolic glycolipid-I (PGL-I) IgM antibodies have been recommended to detect a risk of infection in asymptomatic patients, especially household contacts. Some studies have also suggested a relation between infection in these patients and other biomarkers such as IL-6 and nutritional status (6), serum levels of IgA antibodies against NDO-HSA (7), CCL2 chemokine associated with IFN-γ (8), and IgM profile against NDO-HSA, LID-1, and NDOLID antigens, and monocytes and CD4+ lymphocyte frequency (9), beyond arginase activity (10) as a protective marker against this infection.

Leprosy household contacts present a risk for the development of the disease (11) and could maintain the spread of the *M. leprae* even if the index case is treated since some studies have shown positive PCR for *M. leprae* DNA in samples as nasal swabs, nasal turbinate biopsies, and/or peripheral blood in asymptomatic cases (12–15). Considering that positive results for anti-PGL-I IgM in these household contacts are associated with a higher risk of becoming ill, the evaluation and serology anti-PGL-I IgM of these individuals are recommended (5).

In Brazil, which ranks second worldwide in the number of leprosy’s new cases, MB is the most prevalent form and is associated with neural disabilities in the diagnosis (1). In contrast, the neural involvement in the subclinical infection in these household contacts is still not well elucidated and its evaluation is relevant, especially for the future establishment of chemoprophylaxis protocols.

This study aimed to evaluate the clinical and laboratory predictors of subclinical neural impairment in leprosy household contacts.

## 2. Methods

It is a cross-sectional observational study, from 2017 to 2021, in which we recruited leprosy household contacts from the National Reference Center of Sanitary Dermatology and Leprosy in Brazil, under the approval of the Ethics Committee of the Federal University of Uberlandia. A written informed consent was obtained from all participants for research participation. Some participants were minors and their parents provided written consent on behalf of them.

At this center, leprosy contacts are followed up for a period of at least 7 years, annually, when they are evaluated by a multidisciplinary team and submitted to dermatoneurological examination and serological analyses by Enzyme-linked immunosorbent assay (ELISA) anti-phenolic glycolipid I (anti-PGL-I) Immunoglobulin M (IgM).

**Figure 1:**
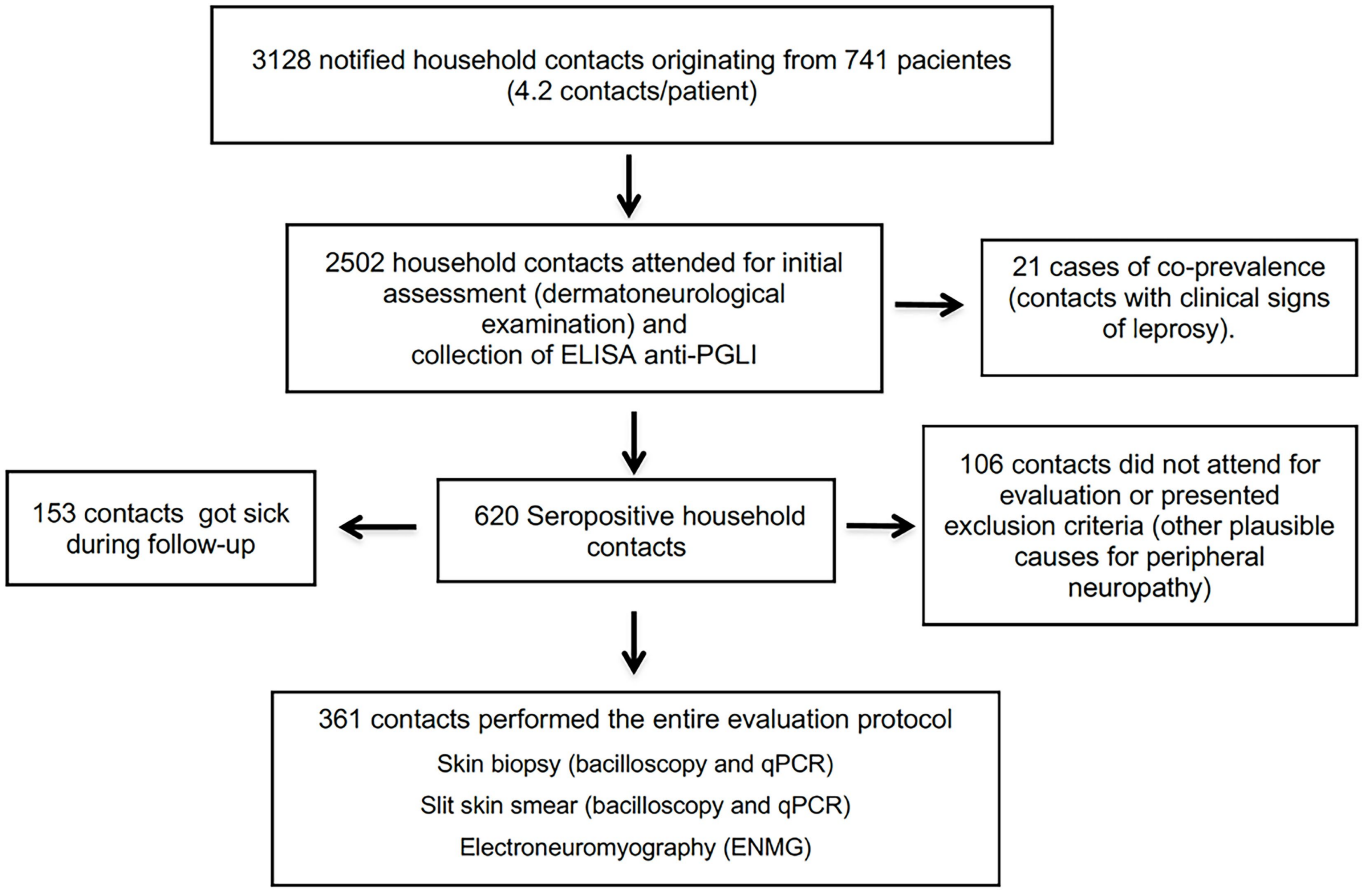
Algorithm proposed for household leprosy contacts selection.

From 2017 to 2021, 741 new cases of leprosy were diagnosed in this service and 3,128 household contacts were notified, totaling an average of 4.2 contacts per patient. A proportion of 77.8% (2,502/3128) of these attended the initial evaluation, when all were submitted to anti-PGL-I serology collection. A total of 21 contacts had clinical signs of leprosy at baseline and 25% (620/2481) were seropositive. In this study, 361 seropositive contacts were submitted to all complementary exams at the time when seropositivity to the anti-PGL-I ELISA was confirmed (Figure 1). We excluded those who showed clinical evidence of leprosy or had any type of neurological symptoms and those who presented other etiologies of peripheral neuropathies, such as: chronic alcoholism, diabetes mellitus, thyroid disease, hormonal dysfunctions, malnutrition, hereditary neuropathy, hepatitis B or C, HIV, autoimmune diseases.

### 2.1. Clinical characterization

Epidemiological and clinical data were recorded. All patients underwent a rigorous dermatoneurological evaluation by two expert professionals (neurologist and dermatologist/leprologist).

### 2.2. Laboratory analyses

Identification of acid-fast bacilli (AFB) – This analyses were performed on slit skin smears from six sites (two ear lobes, two elbows, two knees), as well as skin and/or nerve biopsy samples.

ELISA anti-PGL-I IgM serology – It was performed on all household contacts. Serum anti-PGL-I IgM antibodies were detected by ELISA performed against the purified native PGL-I from the *M. leprae* cell wall. The reagent was obtained through BEI Resources, NIAID, NIH: Monoclonal Anti-*Mycobacterium leprae* PGL-I, Clone CS-48 (produced *in vitro*), NR-19370. The titration of anti-PGL-I antibodies was expressed as an ELISA index according to the proportion between the bacillary load of the sample in relation to the cutoff point. Values above 1.0 were considered positive (16).

DNA Extraction and Real Time Quantitative Polymerase Chain Reaction (qPCR) of the following samples: 1- slit skin smear (one sample) from six sites (two ear lobes, two elbows, two knees); 2- elbow skin biopsy. The qPCR assay targeting *M. leprae* DNA was performed by targeting the bacillus-specific genomic region (RLEP) in a real-time PCR system (ABI 7300, Applied Biosystems, Foster City, CA, United States) (13, 17, 18).

### 2.3. Electroneuromyography

Electroneuromyographic studies were carried out utilizing the MEB 4200 K (NIHON-KODHEN) device. In the sensory conduction study, the median, ulnar, radial, lateral antebrachial cutaneous, median antebrachial cutaneous, sural and fibular superficial were examined bilaterally. In the motor conduction study, the median, ulnar, common fibular, and tibial bilaterally nerves were examined, supplemented by techniques for focal impairment identification at compression sites often affected in leprosy neuropathy, such as median nerve at the wrist, ulnar nerve at the elbow, fibular nerve at the fibular head and tibial nerve at the ankle. The electroneuromyography (ENMG) was used to define the number of affected nerves and also the pattern of neural impairment (mononeuropathy or multiple mononeuropathy). Basically, reduced compound muscle action potential and sensory nerve action potential amplitudes suggest an axonal impairment of peripheral nerves, while prolonged latencies and/or reduced conduction velocities suggest a demyelinating pattern. All examinations were performed by the same neurologist, with expertise in electroneuromyography and leprosy.

### 2.4. Skin biopsy

All of the leprosy contacts selected did not present any skin lesion. For this reason, the biopsy of a small elbow skin fragment was performed, considering that it is a cold region with possible intradermal neural impairment, and therefore a site often altered in leprosy neuropathy. A wedge-shaped incision was made using a scalpel blade, and a fragment of approximately 1 cm along its greatest length that reached the hypodermis was removed. One part of the skin sample to be sent to the molecular pathology and biotechnology laboratory was wrapped in sterilized aluminum paper and immersed in liquid nitrogen. The other part was sent to the institution’s pathology laboratory in a flask containing 10% buffered formalin, for histopathological evaluation. Fite-Faraco staining was used to investigate *M. leprae.*

### 2.5. Statistical analysis

The Shapiro Wilk test was used to test data normality within groups. The Wilcoxon-Mann–Whitney U Test was carried out, and the Binomial Test was applied for the Study of Dichotomous Variables, with significance defined as *p* < 0.05. To assess the level of agreement between the presence of electromyographic abnormalities and the existence of neural thickening, the kappa coefficient analysis was performed. The value of the Kappa coefficient close to 1 indicates that there is agreement between the evaluations and values below 0.60 indicate inadequate agreement. The statistical software used was GraphPad Prism version 7 (La Jolla, CA, United States).

## 3. Results

In this study, 361 seropositive contacts (SPC) were evaluated, with a mean age of 35.7 years (± 18.1) and with a female predominance (66.2%; 239/361). In relation to the type of exposure, 83.1% (300/361) reported intradomiciliary contact with leprosy patients. The mean anti-PGL-I IgM ELISA index was 2.31(±1.03). In the slit skin smear and skin biopsy analysis, the evaluation by the qPCR showed positivity of 35.5% (128/361) and 25.8% (93/361) respectively, all with negative bacilloscopy (Table 1).

**Table 1:** Epidemiological, clinical, and laboratory characteristics among the household contacts of leprosy patients.

	Seropositive household contacts *n* = 361
*Age*	35.7 ± 18.1
*Sex*	
Male	122 (33.8%)
Female	239 (66.2%)
*Type of contact*	
Intradomiciliary	300 (83.1%)
Extradomiciliary	61 (16.9%)
*Index case*	
Multibacillary	306 (84.8%)
Paucibacillary	55 (15.2%)
*ELISA index*	2.31 ± 1.03
Slit skin smear qPCR	128 (35.5%)
Skin biopsy qPCR	93 (25.8%)
Bacilloscopy	0

Only 14.4% (52/361) of the patients were positive in the molecular evaluation by qPCR of RLEP of slit skin smear and skin biopsy and among the 128 patients with positive results in the slit skin smear, 59.4% (76/128) were negative in the skin biopsy.

Regarding the electroneuromyographic evaluation, 23.5% (85/361) presented neural impairment identified by ENMG. 62.3% (53/85) presented a mononeuropathy pattern and 37.7% (32/85) multiple mononeuropathy. The detailed pattern of the ENMG findings is described in Table 2.

**Table 2:** Distribution of the electroneuromyographic pattern in seropositive household contacts of leprosy patients.

Electroneuromyographic pattern	*n*	%
Sensory axonal mononeuropathy	29	34.1
Focal demyelinating mononeuropathy	24	28.2
Asymmetrical sensory and motor demyelinating neuropathy	15	17.6
Asymmetrical sensory and motor axonal neuropathy with focal slowing of conduction velocity	8	9.4
Asymmetrical sensory axonal neuropathy with focal slowing of conduction velocity	5	5.9
Asymmetrical sensory axonal neuropathy	4	4.8
Total	85	100

The mean number of nerves affected was 2.1 per household contact. The most affected sensory nerves were the ulnar, followed by the superficial fibular and sural and among the motor nerves were the common fibular and ulnar. The nerves most frequently affected are described in Table 3.

**Table 3:** Distribution of peripheral nerves most affected in the electroneuromyographic evaluation of the seropositive household contacts of leprosy patients.

Peripheral nerves	*n*	%
**Sensorial nerves**		
Ulnar	41	22.5%
Superficial fibular	24	13.2%
Sural	16	8.8%
Median	6	3.3%
Superficial radial	6	3.3%
Medial antebrachial cutaneous	4	2.2%
Lateral antebrachial cutaneous	2	1.1%
**Motor nerves**		
Common fibular	37	20.3%
Ulnar (elbow)	29	15.9%
Tibial	14	7.7%
Median	3	1.6%
Total		182 2.1 nerves/contact

Regarding the proportion of electroneuromyographic impairment according to the ELISA index, SPC with values above 4.0 showed a higher proportion of neural impairment (Table 4).

**Table 4:** Proportion of electroneuromyographic impairment according to the ELISA index.

ELISA index	Abnormal ENMG
1.1–2.0	22.5% (41/182)
2.1–3.0	19.8% (27/136)
3.1–4.0	23.4% (11/47)
> 4.1	30.0% (6/20)

The presence of clinical neural thickening was observed in 17.5% (63/361) of SPC and among the 85 household contacts with abnormal ENMG, only 25.9% (22/85) presented neural thickening in the clinical evaluation and the agreement between these methods was weak (Table 5).

**Table 5:** Comparison between clinical examination for detection of neural thickening and electroneuromyographic evaluation of seropositive household contacts of leprosy patients.

		Electroneuromyography							
		Normal		Abnormal		Total			
		*n*	%	*n*	%	*n*	%	Kappa	*p*-value
Neural thickening	Normal	235	65.1	63	17.5	298	82.6	0.132	0.011
	Abnormal	41	11.3	22	6.1	63	17.4		
		276		85		361	100		

For the group of SPC with abnormal ENMG, a higher neural thickening frequency was observed. The positivity of the qPCR in slit skin smears and skin biopsy was also higher in this group (Table 6).

**Table 6:** Distribution seropositive household contacts of leprosy patients according to the electroneuromyographic pattern, and comparisons of proportions.

Parameters	Abnormal ENMG *n* = 85	Normal ENMG *n* = 276	*p*-value
ELISA anti-PGL-1 index	2.41 ± 1.20	2.28 ± 0.98	0.52
Neural thickening	22 (25.9%)	41 (14.9%)	0.0192
Slit skin smear qPCR	40 (47.0%)	88 (31.9%)	0.0106
Skin biopsy qPCR	32 (37.6%)	61 (22.1%)	0.0042

## 4. Discussion

In this study, we measured the prevalence of peripheral neural impairment in asymptomatic SPC, through ENMG evaluation.

From 2014 to 2016, we conducted a study in which 175 seropositive and 35 seronegative contacts were recruited and subjected to an extensive protocol that included clinical, molecular, and electroneuromyographic evaluations (19). This study showed that seropositive contacts presented a 4.0-fold higher chance of neural impairment. Since then, electroneuromyographic evaluation has become routine and has been performed in asymptomatic SPC. This study is a continuation of the previous results presented, but carried out in a more timely manner, reaffirming the importance of neurophysiological assessment of this neglected population.

Regarding other clinical forms, primary neural leprosy is the only one that presents with neural impairment without skin lesions or other clinical manifestations. An electrophysiological study is more sensitive than the clinical exam and previous studies showed that abnormalities in ENMG might be present in a high proportion of asymptomatic leprosy patients (20, 21).

The classical neural impairment of leprosy, defined by a sensory impairment with neural thickening before muscle weakness and deformities (20,22–24), was observed in these SPC. Sensory nerve conduction impairment was the most frequent and the earliest parameter in ENMG evaluation (22–24). In contrast, neural thickening does not show agreement with the electrophysiological evaluation, confirming the need for a combined assessment, since the electrophysiological evaluation does not substitute a detailed clinical examination.

The screening of household contacts with anti-PGL-I is well established in the literature and other biomarkers have been evaluated to assess the risk of developing the disease (6–10). Furthermore, neural thickening and/or qPCR of slit skin smear and skin biopsy show a significant association with neural damage and could be used as biomarkers to initiate the treatment in these asymptomatic patients.

This study corroborates the need to make the approach to asymptomatic contacts in endemic countries more timely. Despite the numerous evidence obtained so far, there is no effective recommendation for chemoprophylaxis or for the treatment of asymptomatic contacts who have evidence of subclinical infection using molecular tools. One of the limitations of the study and a point to be observed in the next ones is the prospective evaluation of asymptomatic contacts submitted to chemoprophylaxis, to prove a reduction in neural damage after its implementation. Therefore, as is already done in other chronic infectious diseases, such as tuberculosis, it is necessary to transform these studies into public health policies, since the only way to advance in leprosy control is through early diagnosis.

Clinical evaluation is undoubtedly very important in the clinical approach to patients and household contacts. However, in a disease as complex as leprosy, which in its early stages can present an indolent and subclinical evolution, serological, molecular and neurophysiological tools are essential to break the disease transmission chain.

## Data availability statement

The original contributions presented in the study are included in the article/supplementary material, further inquiries can be directed to the corresponding author.

## Ethics statement

The studies involving human participants were reviewed and approved by Ethics Committee of the Federal University of Uberlandia. Written informed consent to participate in this study was provided by the participants’ legal guardian/next of kin.

## Author contributions

DS and IG contributed from the research design to analysis of the results and the writing of the manuscript. LG, IB, and TO collected data and organized the database, and wrote the draft of the manuscript. DA and AL performed the statistical analysis and wrote the draft of the manuscript. All authors revised the manuscript.

## Funding

The authors thank the Brazilian funding agencies, Brazilian National Council for Scientific and Technological Development (CNPq) and Foundation for Research Support of the State of Minas Gerais (FAPEMIG), for providing financial support to the National Institute of Science and Technology in Theranostics and Nanobiotechnology – INCT- TeraNano – PhD to LRG (Grant numbers: CNPq-465669/2014–0 and FAPEMIG-CBB-APQ- 03613-17). The funders had no role in study design, data collection and analysis, decision to publish, or preparation of the manuscript.

## Conflict of interest

The authors declare that the research was conducted in the absence of any commercial or financial relationships that could be construed as a potential conflict of interest.

## Publisher’s note

All claims expressed in this article are solely those of the authors and do not necessarily represent those of their affiliated organizations, or those of the publisher, the editors and the reviewers. Any product that may be evaluated in this article, or claim that may be made by its manufacturer, is not guaranteed or endorsed by the publisher.
